# Editorial: Women in the history, culture and sociology of sports: 2022–2023

**DOI:** 10.3389/fspor.2024.1367209

**Published:** 2024-01-22

**Authors:** Lucie Schoch, Sheryl Clark

**Affiliations:** ^1^ Université de Lausanne, Lausanne, Switzerland; ^2^ Goldsmiths, University of London, London, United Kingdom

**Keywords:** women, sport, male-dominated careers, underrepresentation in science, gender bias against women

**Editorial on the Research Topic** Women in the history, culture and sociology of sports: 2022–2023

Sport has often been characterized as a male-dominated domain, a trend that is reflected in various facets of scientific research focused on this subject. First, this is evident in a striking lack of women in sports science research across various disciplines. For instance, women have historically received limited attention in sports and exercise medicine research, as highlighted by Costello et al. (1), who noted a “sexual dimorphism and gender disparity in the current literature” (p. 850). This trend is similarly observed in the field of social sciences, such as in the history of sport where recent research has uncovered a longstanding tendency to ignore women, while, in reality, they have played pivotal roles, not just as athletes and spectators, but also as administrators, workers, decision-makers, and leaders in sports organizations globally (2).

Secondly, the male-dominated nature of sports is also reflected in the historical bias within sports science, where female researchers are underrepresented across all disciplines, especially at higher professorial levels and in leadership positions (3). Despite a slight increase in female first authorship in the last two decades, women remain significantly underrepresented in prominent authorship and editorial board positions in sport sciences (4). The issue of implicit gender bias in sport and exercise medicine is also evident in the composition of conference committees, keynote speaker lists, panels, and other events, where there is an overrepresentation of men (5).

In light of this dual underrepresentation of women both as researchers and within the research itself, this Research Topic stands as a compilation of papers designed to provide a platform for female researchers across all fields of sports and physical activity -constituting a prerequisite for publication- and to showcase works on women in sports. Ultimately, this collection contributes to advancements in both theory and methodology, with a commitment to praxis and catalyzing change across a multidisciplinary range of perspectives including literary, historical, sociological and sports leadership disciplinary lenses.

*Rethinking sports history to include sportswomen in 1900s France* offers both a critical indictment of the exclusion of French sportswomen from institutional sporting structures at the time as well as their ongoing exclusion from historical analyses of sport. Drawing on media reports of press organised events for women, Castan-Vicente's interrogation demonstrates how female participants were presented for commodified spectacle even as reports ridiculed, sexualised and essentialised participants' gender identities. Participants' bids for seriousness and legitimacy were thus undermined by their wider exclusion as they were forced to compete in orchestrated events where classed and racialised exotification further worked to deny their athletic credibility. Crucially, as Castan-Vicente points out, we continue to see these impossible gendered contradictions in contemporary media analyses of sportswomen as well as historical accounts that deligitimise activities such as dance or exhibition wrestling. The analysis is thus a much needed call for a more inclusive sport history.

*Canadian women's experiences in mixed-sex sport: Wheelchair rugby* offers an intersectional analysis of a minoritized group within disability sport who are combatting both sexism and ableism through their participation. Women's complex negotiations within these male dominated settings reveal a process of both adaptation and survival. They recounted norms of verbal aggression within team interactions and a demeaning classification system where being a “no pointer” ensured participation even as it diminished ability. It was not surprising then that women prided themselves on survival in the sport and some even feared the creation of a women's league with the view that it might bring down the level of play. They nevertheless highly valued the heightened independence and team bonding they had experienced in same-sex settings. Their passion and dedication comes across strongly in the narratives, and yet these adaptations also worked to reinforce the masculine norms of the sport, which exclude other potential female participants.

*The Underrepresentation of Women in Sport Leadership in South Africa* explores the intersections of gender and sports ideology and its impact on gender (in) equity in the South African context. Drawing from interviews with administrators, gender activists, and sport-for-development professionals, as well as thematic document analysis, the study highlights persistent patriarchal beliefs limiting progress. Men often act as gatekeepers, and traditional masculine attributes continue to be associated with leadership roles in most sports. The study also shows a disconnect between policies and their implementation, revealing a lack of concerted efforts by African sports leaders to address gender equity effectively. The paper underscores that achieving systemic change is both a gradual process and a formidable challenge unless there is a mainstreaming of a gender agenda to effectively address transformative shifts in sports systems and practices.

Finally, *The 5K run in popular fiction: Reading about parkrun and couch to 5K* delves into the fascination with mass-participation running events, focusing on organizations like parkrun and fitness programs such as Couch to 5K. These initiatives play a pivotal role in facilitating involvement for novice runners and traditionally underrepresented groups in regular exercise, notably women. The study revolves around the thematic analysis of four recent novels, organized around the categories of health promotion, individual transformation, and community building. It explores how running communities are imagined and how it reflects, and thus potentially transmits, certain sets of assumptions and values embedded in real-world communities of practice. The analysis reveals that the novels often resemble quasi-instructional manuals, potentially sharing collective tacit knowledge with an implied running community. The study concludes by posit that these works serve as health promotion tools, helping acquaint aspiring runners with the workings of parkrun and Couch to 5K.
